# Association of Neonatal Morbidities and Postnatal Growth Faltering in Preterm Neonates

**DOI:** 10.3390/healthcare13030235

**Published:** 2025-01-24

**Authors:** Justyna Rogulska, Tanis R. Fenton, Tomasz Szczapa, Katarzyna Wróblewska-Seniuk

**Affiliations:** 1 II Department of Neonatology, Poznan University of Medical Sciences, 61-701 Poznan, Poland; 67404@student.ump.edu.pl (J.R.); tszczapa@ump.edu.pl (T.S.); 2 Doctoral School, Poznan University of Medical Sciences, 61-701 Poznan, Poland; 3 Community Health Sciences, O’Brien Institute of Public Health, Cumming School of Medicine, University of Calgary, Calgary, AB T2N 2T9, Canada; 4 Alberta Children’s Hospital Research Institute, Cumming School of Medicine, University of Calgary, Calgary, AB T2N 2T9, Canada

**Keywords:** newborn, postnatal growth faltering, failure to thrive, risk factors, growth, head circumference, length, preterm infant, very low birth weight

## Abstract

Background/Objectives: Postnatal growth faltering (PGF) is a risk factor for adverse neurodevelopment in very preterm neonates. The aim of this retrospective study was to determine which infants’ baseline characteristics, prenatal risk factors and neonatal morbidities are associated with two definitions of PGF: defined as loss of >2 weight z-scores (severe PGF) or as loss of >1 weight, length, and head circumference z-scores between birth and discharge (complex PGF); Methods: 146 premature newborns (<32 weeks of gestational age, <1500 g) were included in the study. Anonymized data including anthropometric measurements (weight, length, and head circumference), perinatal and neonatal data (demographics, maternal morbidities and previous pregnancies, and neonatal and perinatal morbidities) were extracted from the clinical electronic database. Changes in anthropometric age- and sex-specific z-scores using the Fenton 2013 preterm growth charts were calculated to diagnose severe PGF and complex PGF; Results: The incidence of severe PGF was 11% and complex PGF was 24%. Both PGF definitions were associated with bronchopulmonary dysplasia (BPD), severe retinopathy of prematurity (ROP), longer respiratory support, and longer hospital stay. Severe PGF was associated with surgical necrotizing enterocolitis at 25% vs. 1.5%, *p* = 0.001. Complex PGF was associated with severe brain injury at 51% versus 27%, *p* = 0.007. Complex PGF was more common in newborns born most prematurely, while severe PGF was more common in newborns born small for gestational age (SGA); Conclusions: Both severe and complex PGF are associated with several important neonatal morbidities, which might explain why growth faltering is associated with suboptimal neurodevelopment. Appropriate early identification of faltered growth may influence medical and nutrition interventions which in turn could improve the outcome of very preterm newborns.

## 1. Introduction

Very low birth weight (VLBW) in neonates might result from prematurity and hypotrophy or a combination of these factors. Although the survival rate in newborns with very low birth weight is improving, they are still at greater risk of neonatal morbidities and adverse long-term outcomes, mainly impaired neurodevelopment, compared to their larger peers [1,2,3,4]. The risk of these consequences is inversely related to the gestational age and birth weight [5].

Postnatal growth faltering (PGF) is an additional factor that might contribute to suboptimal neurodevelopment in preterm infants, possibly leading to cognitive and motor delays and behavioural and emotional disturbances. It has been shown that inadequate weight and head circumference (HC) after term age and insufficient progress in weight and HC over infancy are associated with poor neurocognitive performance [6,7,8,9]. Consistent evidence has shown that the growth patterns of preterm infants related to sub-optimal neurodevelopment are the slowest growing infants, and those below the growth chart curves between 6 and 24 months of corrected age [10]. In addition, losses of greater than 2 z-scores in weight during neonatal hospital stays have been noted to have more frequent suboptimal neurodevelopment [11,12].

There is a need in neonatal units to identify early those infants whose growth is faltering so that corrective action can be put in place. Growth analysis is a useful method for assessing fetal and neonatal well-being. Growth charts based on birthweight are commonly used as a reference for evaluating anthropometric measurements in preterm VLBW neonates [13,14], and the aim of clinical practice in neonatal intensive care units (NICUs) is generally to reproduce “in utero” growth rates [15,16].

However, although aggressive nutrition protocols have been implemented in NICUs to achieve recommended nutrient intakes and enhance infants’ weight gain, suboptimal postnatal growth is considered a common problem among VLBW infants [17].

Inadequate postnatal growth is defined and diagnosed in many different ways. Extrauterine growth restriction (EUGR) has been a cross-sectional static attitude that refers to the deficit of weight below a determined centile (10th or 3rd) or z-score of expected growth at a specific time, usually around neonatal intensive care unit discharge [6,16]. However, numerous studies have found that this definition has several shortcomings and does not predict neurodevelopmental outcomes, likely because that measurement does not consider the infants’ birthweights and thus does not quantify growth [6,10,11,18,19]. On the contrary, PGF should be a longitudinal dynamic assessment evaluating serial measurements of the weight centiles or z-score differences between birth and the determined t-time (36 or 40 weeks of postmenstrual age, day of discharge, or 28 days of postnatal age) [20]. Such longitudinal assessment is preferred to determine inadequate growth and identify infant malnutrition since it considers infant growth over time and allows for earlier identification of PGF [6,21]. According to ESPGHAN, in the population of preterm newborns, growth faltering might be diagnosed when an infant’s growth in weight, length, or head circumference slows so that they do not follow approximately parallel to a centile during the period of established growth [22].

Many factors can affect the growth of VLBW infants, including prenatal characteristics [23,24], nutrition [22,25,26,27,28], and neonatal morbidities, such as necrotizing enterocolitis (NEC), bronchopulmonary dysplasia (BPD), and late-onset sepsis (LOS) [23,24,29,30,31].

This retrospective study aimed to determine which infant baseline characteristics, prenatal risk factors, and neonatal morbidities are associated with the outcomes of severe and complex PGF.

## 2. Materials and Methods

This retrospective study was conducted in a single tertiary neonatal care unit at Gynecological-Obstetrical University Hospital in Poznan, Poland. The Institutional Review Board of Poznan University of Medical Sciences approved the study’s design. Data were collected for premature infants born with very low birth weight over four years between 1 January 2018 and 31 December 2021. The inclusion criteria for this study were a birth gestational age of <32 weeks and a birth weight of <1500 g. Infants born with chromosomal or congenital anomalies were excluded.

Anonymized data (from birth until discharge from the hospital) were extracted from the clinical electronic database, which prospectively collected daily data on routine infant care. This included routine growth data: weight, length, and HC. Measurements of growth parameters were carried out by nursing staff according to standard operating procedures and using standardized equipment. Weight was measured with a digital scale to the nearest 1.0 g, and HC measures were taken with a non-stretchable tape measure to the nearest 0.5 cm. Absolute measurements were converted to age- and sex-specific z-scores using the Fenton 2013 preterm growth charts [13]. The changes in z-scores were calculated to diagnose (a) severe PGF: loss of >2 weight z-scores from birth to discharge; and (b) complex PGF: simultaneous loss of >1 weight, HC, and length z-score from birth to discharge.

Based on birth weight percentiles, we also stratified infants into small for gestational age (<10th percentile), appropriate for gestational age (10th–90th percentile) and large for gestational age (>90th percentile).

Additional information collected from the database included demographic data (sex, birth gestational age), information on the perinatal period (Apgar score, anthropometric data), maternal morbidities and previous pregnancies, and information about neonatal morbidities during their stay in the neonatal intensive care unit. The following variables concerning neonatal morbidity were obtained:

- respiratory distress syndrome, defined as respiratory insufficiency soon after birth, caused by pulmonary immaturity and requiring surfactant treatment [32],
- bronchopulmonary dysplasia, defined as oxygen, invasive, or non-invasive ventilatory support required at 36 weeks gestational age [33],
- necrotizing enterocolitis (Stage ≥II according to Bell criteria [34]),
- NEC treated with surgery,
- late-onset sepsis, defined as positive blood culture and clinical signs of sepsis >72 h after birth and through NICU hospitalization [35],
- severe retinopathy of prematurity (Stage ≥3 or laser/antivascular endothelial growth factor treatment [36]),
- severe brain injury defined as grade 3/4 intraventricular hemorrhage according to Papile criteria [37] or periventricular leukomalacia,
- length of respiratory support,
- length of mechanical ventilation
- length of hospital stay

Maternal data included maternal age, parity, mode of delivery, single or multiple births, antenatal steroid use, and maternal morbidities:

- diabetes mellitus defined as abnormal fasting glucose or abnormal glucose tolerance test,
- hypertension (both chronic and pregnancy-induced, blood pressure equal to or greater than 140 mmHg or 90 mmHg diastolic),
- preeclampsia defined as hypertension with proteinuria 300 mg/24 h,
- cholestasis diagnosed based on the presence of pruritus and elevated values of bile acids and aminotransferases,
- hypothyroidism, chronic or diagnosed during pregnancy based on thyroid hormones levels and need for L-thyroxine supplementation,
- hyperthyroidism, chronic or diagnosed during pregnancy based on thyroid hormones levels and need for methimazole or propylthiouracil treatment,
- nicotine exposure, defined as any cigarettes used during pregnancy, based on the maternal questionnaire,
- previous pregnancies (previous miscarriages and preterm deliveries).

Based on the PGF definitions mentioned above, we assessed the risk factors for each PGF definition.

Statistical analysis was performed with Statistica 13.1 for Windows (StatSoft Polska). All data were checked for normality. Normally distributed data (according to the Shapiro–Wilk test) were expressed as means ± SD, and data without normal distributions were expressed as medians with maximal and minimal values. For categorical variables, data were expressed as frequency (percentage). The differences between groups were evaluated using the Student’s t-test and Mann–Whitney U-test. The chi-squared Pearson test, chi-squared Yates test, and Fisher’s exact test were used to compare frequencies in different groups, depending on their size. A *p*-value < 0.05 was considered statistically significant.

## 3. Results

A total of 146 infants born ≤32 weeks’ gestation was included after applying eligibility criteria. Birth characteristics and demographic data of included infants are presented in Table 1.

In the studied cohort, 16 (11%) newborns presented with severe PGF, while complex PGF was diagnosed in 35 (24%) infants.

Newborns with severe PGF more frequently were born SGA and less frequently received antenatal steroid therapy (*p* < 0.05) (Table 2). The morbidities associated with severe PGF were bronchopulmonary dysplasia, late-onset sepsis, severe retinopathy of prematurity, and NEC requiring surgical treatment (Table 3). These infants also required longer respiratory support (median 46.5 vs. 37 days; *p* < 0.05), including mechanical ventilation (7.5 vs. 1 days; *p* < 0.05), when compared to their peers with less weight z-score losses (Table 3).

The mean gestational age of newborns who presented with complex PGF was lower than in the non-PGF group. However, contrary to expectations, infants with complex PGF had higher z-scores of weight, length, and head circumference at birth, and were less likely to be born SGA (Table 2). Complex PGF was associated with such morbidities as respiratory distress syndrome, bronchopulmonary dysplasia, severe brain injury, and severe retinopathy of prematurity (Table 3). Still, they stayed longer on respiratory support, including mechanical ventilation (Table 3).

The maternal morbidities, including hypertension, preeclampsia, diabetes mellitus, cholestasis, and obesity, were not associated with severe PGF in newborns. On the contrary, mothers of newborns with complex PGF were more frequently diagnosed with hypertension during pregnancy (Table 4).

Forty-seven newborns in the study group were born small for gestational age (SGA). We noticed that of this number, 9 (19.1%) developed severe PGF, which differed significantly from those born appropriate for gestational age (AGA). On the contrary, complex PGF was significantly more frequently observed in AGA infants (29.4%) (Table 5).

Based on the available research [11,12,24], a post hoc analysis revealed that the statistical power with an alpha level of 0.05 to detect PGF was close to 100%.

## 4. Discussion

The incidence of PGF in the studied population was 11% if defined as severe and 24% when defined as complex PGF. Our study identified that bronchopulmonary dysplasia and severe retinopathy of prematurity were associated with both analyzed definitions of PGF. We also noted that severe PGF was linked with surgical NEC and late-onset sepsis, while complex PGF was related to severe brain injury and respiratory distress syndrome. Both severe and complex PGF were associated with longer respiratory support, longer mechanical ventilation and longer hospital stay. These observations of associations may or may not be showing cause-and-effect relationships.

Our incidence of severe PGF, defined as the loss of weight z-scores >2, was similar to the results of Marks et al. (10.6%) [29] but lower than in the research by Lima et al. (26%) [24], even though the mean gestational age of infants included was higher (30 ± 2 weeks) than in our study (29 ± 3 weeks). Marks et al. observed that being born at a lower gestational age was a risk factor for PGF defined as the loss of weight z-scores >2 [29], which we did not confirm. However, our study observed that complex PGF, defined as simultaneous loss of >1 z-score of weight, length, and HC z-score, was more frequently diagnosed in infants born at a lower gestational age. The reason for that might be that due to immaturity, less mature infants had more difficulty adapting to the postnatal environment.

Head growth in preterm infants serves as an indicator of brain growth and neurodevelopment [8,9]. Optimal head growth may suggest the absence of important neonatal morbidities [38,39]. Serial assessment of head circumference is important for monitoring neurological health and potential neurodevelopmental deficits [8,9]. It is known from some studies that head growth is often the least affected of the three growth parameters, suggesting that brain growth is prioritized [40]. This phenomenon is known as brain-sparing [40,41,42]. There is also a trend for infants to prioritize weight gain at the expense of length growth [43,44]. That prioritization suggests that newborns in whom head growth and length are impaired, as in complex PGF, are probably those with the most difficult adapting. Poor growth is often considered to be the cause of adverse outcomes, while the causal direction might be the opposite due to suboptimal adaption and/or adverse events leading to both suboptimal outcomes and slower growth [10]. Neonatal morbidities often interrupt the ideal administration of nutrition, resulting in suboptimal growth. This, in turn, presents a challenge for infants to overcome these health issues and leads to a cycle of poor growth and neonatal complications [10].

Although very preterm SGA infants tend, on average, to lose less weight postnatally and then regain their birthweights sooner [45], we observed differences in associations for this group. Being born SGA was identified as a risk factor for severe PGF (19% versus 7%), but it appeared to be a protective factor for complex PGF (14% versus 29%). Although for severe PGF, SGA was identified in this analysis as a risk factor, it was not prevalent since only 19% of SGA infants in our study lost more than 2 weight z-scores. Many researchers have observed that intrauterine growth influences the growth pattern of preterm infants after birth. Elmrayed et al. found in their systematic review that preterm infants born SGA had lower BMI, waist circumference, lean body mass, and height in later life when compared to the non-SGA group, which would confirm some growth differences [46]. In contrast, Beukers et al. observed that term-born children born growth-restricted due to early onset placental insufficiency have height and BMI scores comparable to their age-matched peers in adolescence [47]. Fenton et al. observed that at the age of 36 months corrected age, only a minority of SGA infants still presented with measurements below the WHO growth standard 2nd percentile [45]. These findings together confirm that there are some differences but also similarities in growth patterns of preterm infants between those born SGA compared to those born not SGA.

Postnatal growth faltering may be due to multiple factors. It was observed in many studies that infants with morbidities, including sepsis, bronchopulmonary dysplasia, and brain injury, presented more frequently with PGF and, at the same time, with poor neurodevelopmental outcomes [16,48,49,50,51]. The mechanisms that can lead to both these consequences could include prenatal growth restriction, hypoxia, ischemia, inadequate nutrition, and/or inflammation [48,49,50].

Many researchers observed that the most important neonatal morbidities adversely influencing growth are bronchopulmonary dysplasia, patent ductus arteriosus, late-onset sepsis, anemia, and necrotizing enterocolitis [38,52,53,54,55,56]. They also highlighted that infants experiencing poor growth for a longer time require more respiratory support (mechanical and non-invasive ventilation) and longer hospitalization [38,51,52,53,54,55], which confirms the concept that the etiology of growth faltering is not simply due to inadequate nutrition but is complex and also a consequence of morbidities as well as likely prenatal factors [10].

The mechanism that may link PGF with such morbidities as bronchopulmonary dysplasia or retinopathy of prematurity could be a low level of insulin-like growth factor-1 (IGF-I). Insulin-like growth factors (IGFs) are essential mediators of fetal growth and have also been associated with early postnatal growth following preterm birth [57]. Löfqvist et al. observed that in infants who later developed BPD, IGF-I concentrations during the first weeks after very preterm birth were low, probably due to suboptimal nutrition, work of breathing, severe infection, acidosis, and/or other metabolic disturbances [58]. Interestingly, it has also been observed that low levels of IGF-I impair VEGF signalling and are strongly associated with severe retinopathy of prematurity (ROP) [59]. Premature birth results in low IGF-1 serum levels, and with poor growth, and it continues to be low, which has been associated with poor retinal vessel growth and VEGF accumulation [60]. Once the production of endogenous IGF-1 reaches the threshold, it is thought to activate VEGF to cause proliferation in the retina and ROP development [61,62]. Ingolfsland et al. have shown that greater weight gains (in fat mass, fat-free mass, and percentage of body fat from 32 to 37 weeks) were associated with a lower risk of ≥ stage 2 ROP [63]. ROP predictive models use postnatal growth as an alternate estimation for low IGF-1 levels to assess the risk of retinopathy of prematurity [61,62].

Sepsis is another neonatal comorbidity that may affect neonatal growth through inflammation and/or nutritional deficiencies in critical illness [64]. Flannery et al., in a matched cohort of almost 700 VLBW infants born <32 weeks with sepsis, have shown a higher degree of growth faltering that manifested at least 3 weeks after the infection and persisted until NICU discharge [64]. Starc et al., in a retrospective observational study, found that late-onset sepsis was diagnosed less frequently in neonates growing well vs. those who lost >1 weight z-scores (6.1% vs. 19.6%) [55]. In our study, we observed that late-onset sepsis was associated with severe PGF. We assume that sepsis might lead to postnatal growth faltering due to increased energy expenditure during the inflammatory process and catabolic state in the organism, slowing the growth of lean body mass and fat stores [64]. Sepsis also affects the levels of IGF-1, which is crucial for normal early postnatal growth [65,66].

Some studies show the connection between necrotizing enterocolitis and PGF [52,67]; others underline that poor growth is mainly seen after surgically treated NEC [68]. In the studied population, we have not found an association between NEC stage ≥II and PGF, but we observed that NEC requiring surgery was associated with severe PGF. Growth faltering following surgery in the course of necrotizing enterocolitis is due to intestinal failure, defined as a reduction in gut function below the minimum necessary for the absorption of macronutrients, water, and electrolytes [69].

We have not observed any significant association between most maternal morbidities and PGF. However, hypertension was more frequently diagnosed in women whose newborns presented with complex PGF.

We have limited knowledge of the associations between growth faltering and severe long-term consequences [11,12]. An inappropriate supply of essential nutrients may cause suboptimal brain growth, negatively affecting neurodevelopment [53]. Neonatal morbidities associated with PGF may also adversely influence neurodevelopment in the mechanism of hypoxic episodes (BPD) or inflammation (NEC and sepsis) [48,49,70]. PGF might be associated with severe brain injuries such as severe intraventricular hemorrhage or periventricular leukomalacia. In our population, severe brain injury was associated with complex PGF in which not only weight gain but also head circumference and length increase were lower.

Several studies have observed associations between losses of z-scores in the NICU with suboptimal neurodevelopment [7,48,56,71,72,73], mostly using the imperfect Bayley scales as the outcome [74,75]. Single studies that we are aware of have examined losses of z-scores with neurodevelopmental outcomes and found that losses of >1 z-scores for head circumference [71,72,76], weight [77], or length [78] or >2 z-scores for weight [11,12] were predictive. A large Canadian study found that while changes in z-scores were associated with suboptimal neurodevelopment, the sensitivity and specificity were not high enough to be clinically useful [48]. They also observed that neonatal morbidities may make a larger contribution than postnatal growth to neurodevelopment [48]. More work is needed, using diagnostic assessments (sensitivity and specificity) and, ideally, better measures of neurodevelopment [74,75]. Perhaps the definitive answer about which growth measure is most useful will come from studies that examine which of them is most predictive of suboptimal neurodevelopment.

The main strength of this study is that it is a multi-analysis of morbidities associated with PGF diagnosed using different criteria, which helps to understand the complexity of PGF defined by growth changes over time.

A limitation of our study is that it is retrospective observational research that includes only data from a single tertiary care centre and a relatively small number of infants. Since it was retrospective, the collected anthropometric measurements were performed by different medical personnel, which could lead to inconsistency and some measurement errors. The retrospective character of the study also resulted in limited information about socioeconomic factors, so variables such as maternal education, income, nutritional status and access to health services were not available. Genetic factors, parental anthropometric data and determinants of health (as reflected by income and education) might be influential factors for a child’s growth potential. Moreover, growth outcomes can differ depending on the type and amount of nutrition given to the infant, which was not analyzed in the study. Additionally, due to the retrospective character of the study, we could only focus on the short-term outcomes, not on the long-term impact of growth faltering.

Due to the lack of one standard definition of PGF, it is challenging to diagnose this condition reliably. In some cases, PGF might be overdiagnosed, which could lead to overfeeding infants with short- and long-term adverse effects. A disadvantage of the change of 1 weight z-scores in complex PGF was that this loss is expected for many infants due to the postnatal weight loss phase, so it may over-classify infants as growth faltering [79].

## 5. Conclusions

In conclusion, we did not observe any superiority for either of the analyzed measures of PGF. However, both severe and complex PGF were associated with several important neonatal morbidities, which might explain why growth faltering is associated with suboptimal neurodevelopment.

It would be helpful to have a widely accepted definition of PGF to determine specific cut-off values for neonates with growth faltering that need exceptional nutritional support. One definition would also contribute to developing more reliable nutritional recommendations for this population. Additional studies are needed to identify which PGF definition most strongly predicts suboptimal outcomes and which modifications improve outcomes. Appropriate early identification of faltered growth may influence medical and nutrition interventions, which in turn could improve the outcome of very preterm newborns.

## Figures and Tables

**Table 1:** Characteristics of included infants.

Characteristics	Included Infants (n = 146)
Male sex (%)	75 (51.4)
Multiple births (%)	47 (32.2)
Birth gestational age, mean (SD), wk	29 (2.6)
Birth weight, mean (SD), g	1030 (291)
Birth weight z-score, mean (SD)	−0.6 (1.11)
Weight z-score change birth to discharge (SD)	−1.15 (0.73)
Birth length, mean (SD), cm	37.2 (4.6)
Birth length z-score, mean (SD)	−0.09 (1.63)
Length z-score change birth to discharge (SD)	−1.40 (1.10)
HC, mean (SD), cm	25.4 (2.5)
HC z-score, mean (SD)	−0.70 (1.45)
HC z-score change birth to discharge (SD)	−1.16 (1.02)
Small for gestational age (%)	47 (32.2)
Antenatal steroids (%)	120 (82.2)
Respiratory distress syndrome (%)	118 (80.8)
Bronchopulmonary dysplasia (%)	87 (59.6)
Necrotizing enterocolitis stage ≥II (%)	17 (11.6)
NEC treated with surgery (%)	6 (4.1)
Late-onset sepsis (%)	30 (20.5)
Severe brain injury (%)	48 (32.8)
Severe retinopathy of prematurity (%)	11 (7.5)
Length of hospital stay, median (min/max), days	59 (34/232)
Length of respiratory support, median (min/max), days	38 (22/219)
Length of mechanical ventilation, median (min/max), days	1 (0/64)

**Table 2:** Neonatal and anthropometric data of newborns with and without severe and complex postnatal growth faltering.

Characteristics	Severe PGF			Complex PGF		
	PGF (n = 16)	Non-PGF (n = 130)	*p*-Value	PGF (n = 35)	Non-PGF (n = 111)	*p*-Value
Male sex (%)	10 (62.5%)	65 (50%)	*p* = 0.345 ^1^	18 (51.4%)	57 (51.4%)	*p* = 0.994 ^1^
Multiple births (%)	6 (37.5%)	41 (31.5%)	*p* = 0.630 ^1^	10 (28.6%)	37 (33.3%)	*p* = 0.599 ^1^
Birth gestational age, mean (SD)	28.9 (2.7)	29.0 (2.6)	*p* = 0.842 ^2^	28.0 (2.4)	29.3 (2.6)	*p* = 0.006 ^2^
Birth weight, mean (SD), g	929.8 (309.3)	1043.9 (287.3)	*p* = 0.159 ^2^	996.3 (247.9)	1042.5 (303.3)	*p* = 0.356 ^2^
Birth weight z-score, mean (SD)	−0.6 (1.5)	−0.8 (1.1)	*p* = 0.525 ^2^	−0.2 (1.0)	−0.7 (1.1)	*p* = 0.006 ^2^
Weight z-score change birth to discharge (SD)	−2.5 (0.4)	−1.0 (0.6)	*p* < 0.001 ^2^	−1.7 (0.5)	1.0 (0.7)	*p* < 0.001 ^2^
Birth length, mean (SD), cm	35.3 (4.6)	37.4 (4.6)	*p* = 0.057 ^2^	37.6 (4.3)	37.0 (4.7)	*p* = 0.820 ^2^
Birth length z-score, mean (SD)	−0.7 (2.1)	0.0 (1.6)	*p* = 0.094 ^2^	0.6 (1.4)	−0.3 (1.6)	*p* = 0.002 ^2^
Length z-score change birth to discharge (SD)	−2.0 (1.3)	−1.3 (1.1)	*p* = 0.037 ^2^	−2.2 (0.8)	−1.2 (1.1)	*p* < 0.001 ^2^
HC, mean (SD), cm	24.9 (2.5)	25.5 (2.5)	*p* = 0.053 ^2^	25.1 (2.3)	25.5 (2.6)	*p* = 0.665 ^2^
HC z-score, mean (SD)	−1.5 (1.2)	−1.3 (0.8)	*p* = 0.359 ^2^	−0.9 (0.8)	−1.4 (0.9)	*p* = 0.008 ^2^
HC z-score change birth to discharge (SD)	−2.0 (1.6)	−1.1 (0.9)	*p* = 0.025 ^2^	−2.1 (0.9)	−0.9 (0.9)	*p* < 0.001 ^2^
Small for gestational age	9 (56.3%)	38 (29.2%)	*p* = 0.029 ^1^	6 (17.1%)	41 (36.9%)	*p* = 0.029 ^1^
Antenatal steroids	10 (62.5%)	110 (84.6%)	*p* = 0.029 ^1^	28 (80.0%)	92 (68.5%)	*p* = 0.697 ^1^

^1^ chi-squared Pearson test, ^2^ Student’s *t*-test.

**Table 3:** Neonatal morbidities of newborns with and without severe and complex postnatal growth faltering.

Neonatal Morbidities	Severe PGF			Complex PGF		
	PGF (n = 16)	Non-PGF (n = 130)	*p*-Value	PGF (n = 35)	Non-PGF (n = 111)	*p*-Value
Respiratory distress syndrome (%)	15 (93.8)	103 (79.2)	*p* = 0.164 ^1^	34 (97.1)	84 (75.7)	*p* = 0.005 ^1^
Bronchopulmonary dysplasia (%)	15 (93.8)	72 (55.4)	*p* = 0.003 ^1^	30 (85.7)	57 (51.4)	*p* < 0.001 ^1^
Necrotizing enterocolitis stage ≥ II (%)	4 (25)	13 (10)	*p* = 0.095 ^4^	6 (17.1)	11 (9.9)	*p* = 0.241 ^4^
NEC treated with surgery (%)	4 (25)	2 (1.5)	*p* = 0.001 ^4^	3 (8.6)	3 (2.7)	*p* = 0.149 ^4^
Late-onset sepsis (%)	7 (43.8)	23 (17.7)	*p* = 0.035 ^3^	10 (28.6)	20 (18)	*p* = 0.268 ^3^
Severe brain injury (%)	6 (37.5)	42 (32.3)	*p* = 0.677 ^1^	18 (51.4)	30 (27)	*p* = 0.007 ^1^
Severe retinopathy of prematurity (%)	4 (25)	7 (5.4)	*p* = 0.020 ^4^	6 (17.1)	5 (4.5)	*p* = 0.023 ^4^
Length of hospital stay, median (min/max), days	92 (61/210)	53 (29/232)	*p* < 0.001 ^5^	73.5 (38/232)	50.5 (29/210)	*p* < 0.001 ^5^
Length of respiratory support, median (min/max), days	46.5 (28/114)	37 (22/219)	*p* = 0.021 ^5^	46.5 (28/219)	33 (22/120)	*p* < 0.001 ^5^
Length of mechanical ventilation, median (min/max), days	7.5 (0/57)	1 (0/64)	*p* = 0.034 ^5^	4 (0/57)	1 (0/64)	*p* = 0.013 ^5^

^1^ chi-squared Pearson test, ^3^ chi-squared test with Yates correction, ^4^ Fisher’s exact test, ^5^ Mann–Whitney U-test.

**Table 4:** Maternal morbidities in infants with and without severe and complex PGF.

Characteristics	Severe PGF			Complex PGF		
	PGF (n = 16)	Non-PGF (n = 130)	*p*-Value	PGF (n = 35)	Non-PGF (n = 111)	*p*-Value
Maternal diabetes mellitus	3 (18.8%)	17 (13.1%)	*p* = 0.253 ^4^	5 (14.3%)	15 (13.5%)	*p* = 0.974 ^3^
Preeclampsia	1 (6.3%)	16 (12.3%)	*p* = 0.540 ^4^	6 (17.1%)	11 (9.9%)	*p* = 0.138 ^4^
Hypertension	3 (18.8%)	34 (26.2%)	*p* = 0.580 ^4^	13 (37.1%)	24 (21.6%)	*p* = 0.044 ^3^
Nicotine exposure	0 (0%)	5 (3.8%)	*p* = 0.623 ^4^	1 (2.9%)	4 (3.6%)	*p* = 0.692 ^4^
Cholestasis	1 (6.3%)	11 (8.5%)	*p* = 0.245 ^4^	0 (0%)	3 (2.7%)	*p* = 0.467 ^4^
Hypothyroidism	3 (18.8%)	35 (26.9%)	*p* = 0.549 ^4^	8 (22.9%)	30 (27%)	*p* = 0.788 ^3^
Hyperthyroidism	0 (0%)	1 (0.7%)	*p* = 0.911 ^4^	0 (0%)	1 (0.9%)	*p* = 0.778 ^4^
Previous miscarriages	4 (25%)	31 (23.8%)	*p* = 0.378 ^4^	8 (22.9%)	27 (24.3%)	*p* = 0.896 ^3^
Previous preterm deliveries	1 (6.3%)	10 (7.7%)	*p* = 0.656 ^4^	4 (11.4%)	7 (6.3%)	*p* = 0.206 ^4^

^3^ chi-squared test with Yates correction, ^4^ Fisher’s exact test.

**Table 5:** The association between being small for gestational age and severe and complex postnatal growth faltering.

	Severe PGF		Complex PGF	
	n = 16		n = 35	
SGA (n = 47)	9 (19.1%)	*p* = 0.045 ^4^	6 (13.7%)	*p* = 0.048 ^3^
AGA (n = 99)	7 (7.1%)		29 (29.3%)	

^3^ chi-squared test with Yates correction, ^4^ Fisher’s exact test.

## Data Availability

The corresponding author can be contacted to share the raw data, if based on a reasonable request and study protocol.
